# The epidemiology of alcohol involved facial injuries

**DOI:** 10.1007/s10006-025-01343-5

**Published:** 2025-01-28

**Authors:** Arya Sherafat, Brian Sangalang, Nihal Punjabi, Ian Waldrop, Emily Dubina, Jared C. Inman, Nicholas W. Sheets

**Affiliations:** 1 University of California, Riverside School of Medicine, Riverside, CA USA; 2 Department of Otolaryngology – Head and Neck Surgery, Loma Linda Medical Center, Loma Linda, CA USA; 3 Case Western Reserve University School of Medicine, Cleveland, OH USA; 4 Department of Surgery, Riverside Community Hospital, 4445 Magnolia Ave, Riverside, CA 92501 USA

**Keywords:** Facial injury, Trauma, Craniofacial, Alcohol, Epidemiology

## Abstract

**Purpose:**

Alcohol use has been shown to affect injury patterns and risk of trauma. This study aims to characterize the epidemiologic characteristics of alcohol involved facial injuries presenting to US emergency departments.

**Methods:**

This study reports a cross-sectional analysis of patients with facial injuries within the National Electronic Injury Surveillance System (NEISS). Demographics, disposition, and mechanism of injury were compared between facial injury patients with reported/suspected alcohol consumption prior to or during the time of injury (AIFI+) and facial injury patients with no alcohol consumption (AIFI-).

**Results:**

A total of 37,777 facial injuries were reported within the NEISS. Out if these, 3,336 patients experienced an alcohol involved facial injury (AIFI+). AIFI + patients were younger than AIFI- patients (47 vs. 57, *p* < 0.001), more likely to be male (68.5% vs. 31.5%, *p* < 0.001), and more likely to be White (51.6% vs. 53.6%, *p* = 0.03). Patients with AIFI were less likely to be injured at home (41.5% vs. 45.5%, *p* < 0.001) and more likely to be injured in the street (8.5% vs. 4.5%, *p* < 0.001). Disposition differed with AIFI + patients less likely to be treated and released (78.8% vs. 83.3%, *p* < 0.001) and more likely to leave without being seen (3.8% vs. 1.8%, *p* < 0.001).

**Conclusions:**

Our study reports that AIFI + patients are younger, more likely to be injured on the street, and more likely to be injured by stairs than AIFI- patients. Additionally, patients with an AIFI + are more likely to leave the hospital without being seen.

**Supplementary Information:**

The online version contains supplementary material available at 10.1007/s10006-025-01343-5.

## Introduction

Previous reports have found that approximately 25% of all traumas involve the face [1, 2]. Facial injuries can result in impaired sensory perception, communication, and altered aesthetics. Additionally, facial trauma of the head and neck (fractures, lacerations, contusions, and abrasions) can have physical and psychosocial consequences, such as posttraumatic stress disorder and alcohol use disorder [3, 4]. Alcohol use and binge drinking have been linked to a high prevalence of trauma [5–9]. A study examining the effect of alcohol consumption on overall injury rates found that alcohol use was associated with a 3.4-fold increase in the likelihood of injury [10]. The prevalence of facial injuries has been rising in recent years [11]. While previous research has already examined alcohol involved facial injuries caused by interpersonal violence and motor vehicle accidents [12, 13], few studies have directly investigated the relationship between alcohol consumption and patterns of facial injury beyond these etiologies. Therefore, it is important to describe the differences in characteristics, disposition, and place of injury between alcohol involved facial injuries (AIFI+) and non-alcohol involved facial injuries (AIFI-) to optimize treatment for this sub-population and guide targeted public health measures to prevent future injuries. This study aims to compare the epidemiologic characteristics between AIFI + and AIFI- patients caused by common consumer products.

## Methods

This study was IRB exempt because it utilized existing data that are publicly available and deidentified. Consent was not collected since all patients represented were from a deidentified database. Research was conducted in accordance with the Declaration of Helsinki.

### Data source

This study reports a cross-sectional analysis of patient data from the National Electronic Injury Surveillance System (NEISS) collected by the Consumer Product Safety Commission (CPSC) from January 1, 2019, to December 31, 2022, in the United States. The NEISS is a stratified probability sample of over 100 US hospital emergency departments. The national prevalence of facial injuries and mechanisms by which these injuries occur can be extrapolated from the NEISS data. The NEISS includes information extracted from medical charts, including patient demographics (i.e., age, sex, and race) and injury information including body part injured, diagnosis, geographic location where the injury occurred, product involved, and a narrative of the injury event. Facial Injuries were categorized by primary diagnosis code 76, which reports injuries of the face, including the eyelid, eye area, nose, and forehead.

### Variables

This study included all patients reported within the NEISS 18 years of age or greater who had a facial injury primary diagnosis (code 76) between 2019 and 2022. The study sample was further categorized by facial injuries with reported/suspected alcohol use and facial injuries with no reported alcohol use. The NEISS reports alcohol use if the patient consumed alcohol prior to or during the incident, if there is a positive BAL/BAC/breath test, or if there is suspected alcohol use but no breath tests available. Comparative analyses were performed to assess differences in the epidemiologic characteristics of facial injuries in patients with suspected/reported alcohol use (AIFI+) compared to facial injuries in patients with no reported/suspected alcohol use (AIFI-).

The products that included 95% of facial injuries were categorized in accordance with broadly shared characteristics: activities (i.e. fishing, exercise, sports), elevated steps (i.e. stairs, ladders, stepstools), finishings (i.e. furniture, cabinets), recreational vehicles (i.e. motorized or unmotorized skateboards, bikes, ATVs), sharps (i.e. glass, knives, power tools), surfaces (i.e. walls, ceilings, floors), and tripping hazards (i.e. pet toys, rugs). However, exact mechanism of injury (i.e. accidental vs. non-accidental) was not differentiated in the data base Licensed motorized vehicles (cars, motorcycles, RVs, helicopters, etc.) that are used for ground transportation and gun violence were not available in the NEISS database.

### Statistical analysis

The NEISS is a probability sample of all hospitals with emergency departments in the US, therefore analyses were performed accounting for statistical weights. Statistical weights provided by the CPSC were used to calculate national injury estimates. Microsoft excel (2023) was used for all analyses, and *P*-values < 0.05 were considered statistically significant. For all calculated estimates, a 95% confidence interval (CI) was determined, accounting for sampling error, as outlined by the CPSC.

Demographic and facial injury characteristics were compared between AIFI- and AIFI + groups using Chi-squared tests. Additionally, demographic data (age, sex, race) were represented as percentiles by subdividing the population based on identifying characteristics. Relative Risk (AIFI+/AIFI-) was calculated for the top 25 most common products in each group.

## Results

### Trends in number of AIFI by patient demographics

**Fig. 1:**
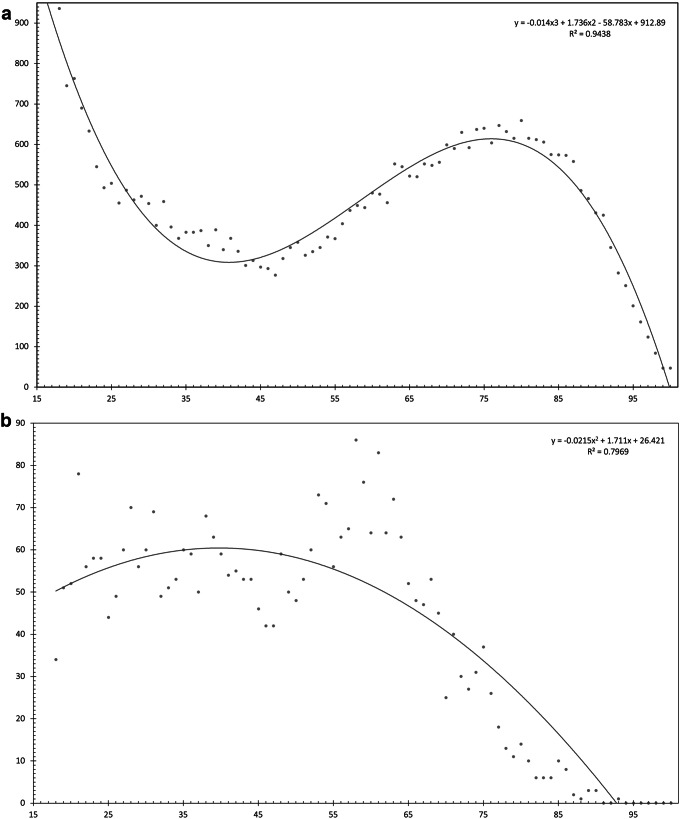
**A**. Age vs. number of injuries in the AIFI- group with a polynomial equation fitted to the data. **B**. Age vs. number of injuries in the AIFI + group with a polynomial equation fitted to the data

Of the 37,777 facial injuries reported in the NEISS, 3,336 (0.09%) were AIFI+. This represents a national estimate of 144,516 (95% CI: 125,417–163,616) AIFI in the United States between 2019 and 2022. The yearly incidence of AIFI did not significantly differ within the study period, with an estimated mean of 36,129 (95% CI: 26,606 − 45,652) AIFI reported annually. AIFI + patients were younger than AIFI- patients (47 years vs. 57 years, *p* < 0.05). AIFI- patients had a bimodal distribution of injuries with respect to age with a larger peak in very young patients, and another peak around age 80 (Fig. 1A). The prevalence AIFI + injuries was relatively constant for all ages below 55, followed by a slight peak between ages 55–65, and then a sharp decline after age 65 (Fig. 1B). Men experienced AIFI more frequently than women (M 68.5% vs. F 31.5%, *p* < 0.05). The majority of AIFI + patients were White (51.7%) followed by Black/African American (17.1%).

### Differences in AIFI’s by injury type and place

The most common facial injuries among both groups were lacerations (AIFI + 34.2% vs. AIFI- 38.4%, *p* < 0.001) followed by contusions/abrasions (AIFI + 19.9% vs. AIFI- 28.1%, *p* < 0.001). Facial fractures constituted the third most common type of injury in both groups (AIFI + 15.3% vs. AIFI- 16.0%, *p* = 0.1). Patients with an AIFI were less likely to be injured at home (AIFI + 41.5% vs. AIFI- 45.5%, *p* < 0.05) and more likely to be injured in the street (AIFI + 8.5% vs. AIFI- 4.5%, *p* < 0.001).

### Disposition of AIFI presenting to emergency departments

A majority of both AIFI + and AIFI- groups presented to “Very Large” Emergency Departments (AIFI + 46.2% vs. AIFI- 48.6%, *p* < 0.01). Beyond “Very Large” emergency departments, AIFI + injuries more commonly presented to “Large” Emergency Departments (*p* < 0.001) while AIFI- injuries were more commonly treated at “Small, Medium, Very Large, or Children’s” Emergency Departments (*p* < 0.001, *p* < 0.001, *p =* 0.01, and *p* < 0.001 respectively).

**Fig. 2:**
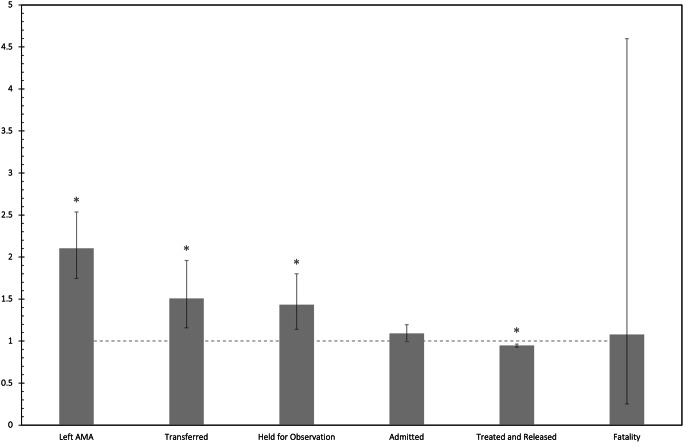
Relative risk (AIFI+/AIFI-) for disposition status with 95% confidence intervals. Dashed line represents the equation y = 1. * = *p* < 0.05

AIFI + patients had a 2.1 times greater risk of leaving the emergency department against medical advice (RR = 2.10, *p* < 0.001). AIFI + patients also had a 1.51 times greater risk of being transferred to another facility and a 1.43 times greater risk of being held for observation. AIFI + patients were less likely to be treated and discharged from the emergency department in comparison to AIFI- patients (RR = 0.95, *p* < 0.001)(Fig. 2).

### Differences in AIFI’s by products involved in injury & grouped products

The top 3 products involved in AIFI + patients’ injuries were stairs, floors, and bicycles (20.48%, 18.14%, and 7.04% respectively) while the top 3 products in AIFI- patients’ injuries were floors, beds, and stairs (20.75%, 8.33%, and 6.92% respectively) (Table 1). Alcohol involvement led to a 2.96 times greater risk of facial injury related to stairs and a 2.87 times greater risk of facial injury related to motorized scooters. Alcohol was also associated with a higher risk of facial injury involving bicycles, porches, fences, tables, walls, and counters. Alcohol involvement was associated with a decreased risk of facial injury related to beds, toilets, and cabinets (RR = 0.38 *p* < 0.001, RR = 0.51 *p* < 0.001, and RR = 0.54 *p* < 0.001 respectively). Alcohol was also associated with a decreased risk of facial injury involving floors, rugs, doors, footwear, desks, and bathtubs. A complete list of all products reported in the NEISS with frequency counts is presented in supplementary table S1.

**Fig. 3:**
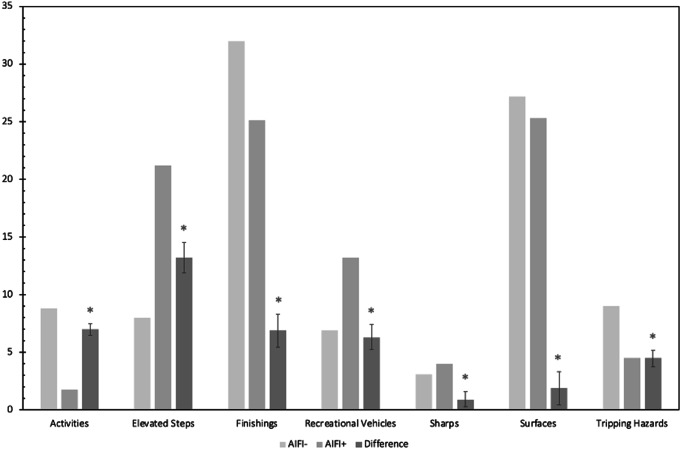
Products involved in 95% (2 SD) of injuries categorized by type. Standard error bar represents 95% confidence interval. * = *p* < 0.05

All categorized products showed a significant difference (*P* < 0.05) in mechanism of injury between AIFI + and AIFI- patients. Patients who were in the AIFI + group had a higher number of injuries resulting from elevated steps, recreational vehicles, and sharps compared to AIFI- patients. Alternatively, AIFI- patients had a higher number of injuries resulting from activities, home finishings, surfaces, and tripping hazards (Fig. 3).

**Table 1:** Comparison of most common products involved in facial injuries. The top 17 products were involved in > 66% (1 SD) of the injuries in both AIFI- and AIFI + groups. RR (AIFI+/AIFI-) was calculated for the top 25 most common products in each group. Only significant results are presented. For a full list of all 500 + included products with frequency counts, refer to supplementary table S1

AIFI- (%)		AIFI+ (%)		RR (95% CI)		
Floors	20.75	Stairs	20.48	Stairs	2.96 (2.76, 3.18)	*P* < 0.0001
Beds	8.33	Floors	18.14	Beds	0.38 (0.32, 0.46)	*P* < 0.0001
Stairs	6.92	Bicycles	7.04	Bicycles	1.84 (1.62, 2.08)	*P* < 0.0001
Bicycles	3.83	Tables	4.41	Scooters	2.87 (2.23, 3.68)	*P* < 0.0001
Tables	3.72	Walls	3.24	Toilets	0.51 (0.37, 0.69)	*P* < 0.0001
Chairs	2.92	Beds	3.19	Porches	1.93 (1.51, 2.46)	*P* < 0.0001
Walls	2.66	Chairs	2.68	Fences	1.83 (1.36, 2.46)	*P* = 0.0001
Bathtubs	2.44	Scooters	1.99	Floors	0.87 (0.82, 0.94)	*P* = 0.0002
Toilets	2.20	Porches	1.97	Cabinets	0.54 (0.38, 0.77)	*P* = 0.0007
Doors	2.06	Bathtubs	1.86	Rugs	0.59 (0.43, 0.81)	*P* = 0.0011
Basketball	1.89	Doors	1.43	Doors	0.70 (0.53, 0.91)	*P* = 0.0091
Rugs	1.77	Counters	1.33	Footwear	0.67 (0.49, 0.93)	*P* = 0.0159
Cabinets	1.56	Fences	1.30	Desks	0.65 (0.45, 0.93)	*P* = 0.0188
Footwear	1.54	Mopeds	1.30	Bathtubs	0.76 (0.60, 0.97)	*P* = 0.0267
Exercise	1.38	Toilets	1.12	Tables	1.19 (1.02, 1.39)	*P* = 0.0306
Desks	1.24	Barstools	1.09	Walls	1.22 (1.01, 1.46)	*P* = 0.0341
Porches	1.02	Rugs	1.04	Counters	1.35 (1.01, 1.80)	*P* = 0.0427

### Differences in AIFI’s by top products involved in injury & injury type

Our data shows significant increases in AIFI + lacerations compared to AIFI- lacerations that happened on stairs (49.55% vs. 47.08%, *p* < 0.01)) and Table (79.22% vs. 61.19%, *p* < 0.01). Additionally, hematomas in the AIFI + group were generally greater then AIFI- hematomas with significant increases occurring with stairs (8.16% vs. 6.49%, *p* < 0.01) and floors (13.68% vs. 9.50%, *p* = 0.01). AIFI + contusions on stairs were significantly less than the AIFI- group (10.39% vs. 23.59%, *p* = 0.01). AIFI + fractures on stairs and floors were less than the AIFI- fractures in the same category (12.69% vs. 18.02%, *p* = 0.02 & 4.86% vs. 12.26%, *p* < 0.01, respectively), while AIFI + fractures with walls were significantly greater than AIFI- fractures with walls (17.54% vs. 8.45%, *p* = 0.04) (Table 2).

**Table 2:** Comparison of top 5 products involved in AIFI injury to top 4 injury types using chi-squared tests. Significant p-values are bolded. Contusions category includes abrasions

		Laceration	Contusion	Fracture	Hematoma
Stairs	AIFI +	49.55%	26.59%	12.69%	8.16%
	AIFI -	47.08%	26.56%	18.02%	6.49%
	p-value	**< 0.01**	0.95	**0.02**	**< 0.01**
Floors	AIFI +	37.69%	37.39%	4.86%	13.68%
	AIFI -	40.86%	36.19%	12.26%	9.50%
	p-value	0.27	0.70	**< 0.01**	**0.02**
Bicycles	AIFI +	52.94%	20.59%	20.59%	5.88%
	AIFI -	51.46%	24.16%	20.08%	3.56%
	p-value	0.99	0.43	0.92	0.39
Tables	AIFI +	79.22%	10.39%	5.19%	5.19%
	AIFI -	61.19%	23.59%	8.45%	6.01%
	p-value	**< 0.01**	**0.01**	0.43	0.96
Walls	AIFI +	45.61%	26.32%	17.54%	8.77%
	AIFI -	53.54%	28.93%	8.45%	8.13%
	p-value	0.30	0.78	**0.04**	0.94

## Discussion

From 2019 to 2022, an estimated 5 million injuries involving the facial region presented to United States Emergency Departments as determined from the *National Electronic Injury Surveillance System* (NEISS). Of these injuries, an estimated 144,516 were alcohol involved facial injuries. As alcohol use and binge drinking continue to be widespread across the United States [14, 15], characterization of the epidemiology of AIFI compared to non-alcohol involved facial injuries (AIFI-) is important to highlight special considerations when caring for this sub-population in emergency departments.

### Demographics

Our study demonstrates a bimodal distribution in age for AIFI- patients, with increased prevalence in young adults (age 18–22) and older adults (age 76–80) (Fig. 1A). This might be because young adults are more likely to participate in contact sports [16], while older adults are at greater risk for fall injuries [17, 18]. Amongst AIFI- patients, middle-aged adults had the lowest number of injuries. In contrast, middle-aged adults (age 55–65) had the highest prevalence out of all AIFI + patients (Fig. 1B). A 2019 study reported that the prevalence of heavy alcohol use was greatest in middle/older aged adults between the ages of 55–70 [19]. Our study is consistent with these findings, as AIFI were observed the most amongst this age range. Alcohol use in middle/older age adults is thought to be rooted in generational social norms associated with drinking, more spare time to drink, and fewer responsibilities [19–21].

Although gender gaps in patterns of alcohol consumption are narrowing [22], our data shows a higher prevalence of AIFI + in men. These results are consistent with previous studies which conclude that facial trauma is most prevalent amongst men 20–40 years of age [23, 24]. Racial differences were also observed, with White patients experiencing the highest overall prevalence of AIFI+. However, Black patients were overrepresented in the AIFI + group, making up 17.1% of AIFI + injuries, while recent national estimates show that only 13.6% of the population is Black. On the other hand, White patients were underrepresented compared to the national population (51.7% vs. 75.5%). A systematic review conducted by Keyes et al. on the interaction of race and alcohol-attributable injuries had similar findings despite lower alcohol use in Black populations. Some possible explanations they suggest for this pattern are that alcohol consumption may be underreported in minorities, differences in neighborhood infrastructure may make certain environments more likely to cause injury, and racial bias in the assessment of alcohol positivity may lead to an overestimation of minority patients in the alcohol positive group [25].

### Disposition

While AIFI- patients were more likely to be treated at small, medium, and very large emergency departments, AIFI + patients were more likely to be treated at large emergency departments. AIFI + patients might need more resources for treatment, which larger clinical settings are able to offer. Additionally, patients with alcohol related hospitalization typically spend more time in inpatient settings and have a longer length of stay [26–27]. This trend is further supported by our data showing that AIFI + patients had a lower likelihood of being treated and discharged from the emergency department compared to AIFI- patients.

AIFI + patients also had a greater risk of leaving the ED against medical advice (AMA), being transferred to another facility, and being held for observation (Fig. 2). All these outcomes are associated with increased healthcare costs and utilization of resources. Patients who leave the ED AMA have especially been shown to have higher healthcare costs, worse outcomes, and readmission rates up to 4x greater than those who do not leave AMA [28–31]. Therefore, hospital personnel should receive additional training in supporting intoxicated patients to prevent them from leaving without appropriate treatment (i.e. consulting social work earlier).

### Products

The most common product involved in AIFI + injuries were stairs and steps compared to floors and flooring materials in AIFI- injuries (Table 1). Considering that AIFI + patients experienced a higher number of injuries in public spaces, stairs located on sidewalks or streets might pose a higher risk to developing an AIFI. Our data shows that AIFI + patients also had a higher risk of developing injury from motorized scooters, which supports a previous study that reported this phenomenon in facial fractures [32].

### Grouped products & place of injury

Patients with an AIFI had a higher prevalence of injury resulting from an elevated fall, recreational vehicles, and sharps compared to AIFI- patients (Fig. 3). With alcohol use, the fine motor coordination necessary to operate a vehicle or properly handle sharps (knives, power tools, etc.) is diminished. Middle-aged/older adults, who had the highest number of AIFI in our data, are especially impaired by alcohol [33]. Alternatively, AIFI- patients had a higher prevalence of injuries resulting from activities, finishings, surfaces, and tripping hazards. In the AIFI- group, injuries were more likely to occur at home. Considering that at-home injuries cause an estimated > 30,000 deaths and 12 million nonfatal injuries annually in the United States [34], it is important to establish safety education programs for patients who might be at high-risk for at-home hazards. This data might also reflect increasing rates of stay-at-home lifestyles throughout/following the COVID-19 pandemic [35, 36].

In contrast, our study demonstrates that AIFI + patients were less likely to be injured at home and more likely to be injured on the street. This result supports a previous study that demonstrated an association between alcohol consumption and public/street injury locations [37].

Top products and Injury type.

Stair injuries resulted in significantly more lacerations and hematomas in AIFI + than AIFI- patients. AIFI + fractures, however, were significantly lower than the AIFI- group. The older age of the AIFI- group likely explains this result, since bone density decreases with age [38]. This trend was also observed in floor injuries, with more fractures occurring in AIFI- patients. There were also significantly more lacerations involving tables in the AIFI + group, which is supported by a previous study concluding that 25% of furniture-related injuries result from tables, and most of these injuries were lacerations to the head and neck [39]. The addition of alcohol to the already high prevalence of table lacerations explains these results.

### Limitations and future directions

The major limitation of this study is that only patients who sought medical care in the emergency setting were included. Therefore, those who experienced facial injuries but did not seek medical care are not represented within this study. Additionally, the NEISS only began recording alcohol involvement beginning in 2019 and has data up to 2022 at the time this study was conducted. This 4-year period might be limited in providing long-term conclusions, and results may have been impacted by the COVID-19 pandemic. Differences in reporting alcohol involvement may have been variable between practitioners. Although the NEISS has guidelines as to what alcohol involvement is reported, these differences may influence the accuracy of the study samples alcohol involvement. Lastly, injury resulting from licensed motorized vehicles was not included in this study, as it is recorded by the Department of Transportation and not the CPSC. Future research should study licensed motorized vehicles and alcohol involved facial injuries using large, nationally representative databases. Additionally, research on safety measures to help prevent injuries in public spaces and stairs is needed.

## Conclusion

Our study reports that AIFI + patients are younger, more likely to be injured on the street, and more likely to be injured by stairs than AIFI- patients. Although most AIFI + patients were White, Black patients were over-represented in the AIFI + group when compared to national demographic estimates. AIFI + patients also had more injuries resulting from elevated steps, recreational vehicles, and sharps. Additionally, AIFI + patients were more likely to leave the hospital without being seen. Understanding the prevalence, causes, demographics, and outcomes of alcohol involved facial injuries can help design effective injury prevention programs, improve trauma care protocols, and reduce the overall burden of these injuries. Characterization of AIFI will allow emergency departments and healthcare personnel to optimize treatment for this patient population.

## Electronic supplementary material

Below is the link to the electronic supplementary material.

Supplementary Material 1

## Data Availability

Data from the National Electronic Injury Surveillance System used in this analysis can be accessed at https://www.cpsc.gov/Research--Statistics/NEISS-Injury-Data.
